# MYB308-mediated transcriptional activation of plasma membrane H^**+**^-ATPase 6 promotes iron uptake in citrus

**DOI:** 10.1093/hr/uhac088

**Published:** 2022-04-11

**Authors:** Zhengyan Fan, Yifang Wu, Liuying Zhao, Lina Fu, Lile Deng, Jiarui Deng, Dekuan Ding, Shunyuan Xiao, Xiuxin Deng, Shu’ang Peng, Zhiyong Pan

**Affiliations:** Key Laboratory of Horticultural Plant Biology (Ministry of Education), Key Laboratory of Horticultural Crop Biology and Genetic Improvement (Central Region, Ministry of Agriculture), College of Horticulture and Forestry Sciences, Huazhong Agricultural University, Wuhan 430070, China; Key Laboratory of Horticultural Plant Biology (Ministry of Education), Key Laboratory of Horticultural Crop Biology and Genetic Improvement (Central Region, Ministry of Agriculture), College of Horticulture and Forestry Sciences, Huazhong Agricultural University, Wuhan 430070, China; Key Laboratory of Horticultural Plant Biology (Ministry of Education), Key Laboratory of Horticultural Crop Biology and Genetic Improvement (Central Region, Ministry of Agriculture), College of Horticulture and Forestry Sciences, Huazhong Agricultural University, Wuhan 430070, China; Key Laboratory of Horticultural Plant Biology (Ministry of Education), Key Laboratory of Horticultural Crop Biology and Genetic Improvement (Central Region, Ministry of Agriculture), College of Horticulture and Forestry Sciences, Huazhong Agricultural University, Wuhan 430070, China; Key Laboratory of Horticultural Plant Biology (Ministry of Education), Key Laboratory of Horticultural Crop Biology and Genetic Improvement (Central Region, Ministry of Agriculture), College of Horticulture and Forestry Sciences, Huazhong Agricultural University, Wuhan 430070, China; Chenggu Fruit Industry Technical Guidance Station, Shaanxi 723200, China; Chenggu Fruit Industry Technical Guidance Station, Shaanxi 723200, China; Institute for Bioscience and Biotechnology Research & Department of Plant Sciences and Landscape Architecture, University of Maryland College Park, Rockville, MD 20850, USA; Key Laboratory of Horticultural Plant Biology (Ministry of Education), Key Laboratory of Horticultural Crop Biology and Genetic Improvement (Central Region, Ministry of Agriculture), College of Horticulture and Forestry Sciences, Huazhong Agricultural University, Wuhan 430070, China; Key Laboratory of Horticultural Plant Biology (Ministry of Education), Key Laboratory of Horticultural Crop Biology and Genetic Improvement (Central Region, Ministry of Agriculture), College of Horticulture and Forestry Sciences, Huazhong Agricultural University, Wuhan 430070, China; Key Laboratory of Horticultural Plant Biology (Ministry of Education), Key Laboratory of Horticultural Crop Biology and Genetic Improvement (Central Region, Ministry of Agriculture), College of Horticulture and Forestry Sciences, Huazhong Agricultural University, Wuhan 430070, China

## Abstract

Iron-deficiency chlorosis is a common nutritional disorder in crops grown on alkaline or calcareous soils. Although the acclimation mechanism to iron deficiency has been investigated, the genetic regulation of iron acquisition is still unclear. Here, by comparing the iron uptake process between the iron-poor-soil-tolerant citrus species Zhique (ZQ) and the iron-poor-soil-sensitive citrus species trifoliate orange (TO), we discovered that enhanced root H ^+^ efflux is crucial for the tolerance to iron deficiency in ZQ. The H^+^ efflux is mainly regulated by a plasma membrane-localized H^+^-ATPase, HA6, the expression of which is upregulated in plants grown in soil with low iron content, and significantly higher in the roots of ZQ than TO. Overexpression of the *HA6* gene in the *Arabidopsis thaliana aha2* mutant, defective in iron uptake, recovered the wild-type phenotype. In parallel, overexpression of the *HA6* gene in TO significantly increased iron content of plants. Moreover, an iron deficiency-induced transcription factor, MYB308, was revealed to bind the promoter and activate the expression of *HA6* in ZQ in yeast one-hybrid, electrophoretic mobility shift, and dual-luciferase assays. Overexpression of *MYB308* in ZQ roots significantly increased the expression level of the *HA6* gene. However, MYB308 cannot bind or activate the *HA6* promoter in TO due to the sequence variation of the corresponding MYB308 binding motif. Taking these results together, we propose that the MYB308 could activate *HA6* to promote root H^+^ efflux and iron uptake, and that the distinctive MYB308-*HA6* transcriptional module may be, at least in part, responsible for the iron deficiency tolerance in citrus.

## Introduction

Iron is an essential micronutrient for plant growth, development, and reproduction [1]. Although the total iron content in the earth’s crust is generally sufficient, iron bioavailability is very poor, particularly in alkaline and/or calcareous soils. In such soils, most crops suffer from iron-deficiency chlorosis, which could lead to reduction in crop yield and quality [2]. Citrus is one of the most important fruit crops in the world in terms of production, and most citrus are cultured in hilly areas with barren soil and they are sensitive to iron deficiency and display severe leaf/shoot chlorosis [3, 4]. Therefore, iron deficiency is one of the major abiotic stresses limiting the productivity, quality, and geographical distribution of citrus [5].

Plants have evolved two different strategies aimed at improving iron acquisition from the rhizosphere. Dicotyledonous plants such as *Arabidopsis thaliana* have evolved a reduction-based strategy (Strategy I) for iron acquisition. In Strategy I plants, at first the Fe^3+^ complexes from soil are solubilized through H^+^ efflux-mediated rhizosphere acidification; second, the resulting Fe^3+^ is reduced to Fe^2+^, a process that depends on FERRIC REDUCTION OXIDASE 2 (FRO2) [6]; third, the Fe^2+^ is subsequently transported into root cells by the divalent metal transporter IRON-REGULATED TRANSPORTER 1 (IRT1) [7]. By contrast, Strategy II plants, represented by graminaceous monocots, directly take up Fe^3+^ by secreting Fe^3+^-chelating substances [8].

Rhizosphere acidification is a prerequisite step for iron uptake because iron solubility decreases ~1000-fold with each unit increase in soil pH within a range of pH 4–9 [9]. The step is achieved by plasma membrane (PM) H^+^-ATPases (HAs), which are functional ~100-kDa monomers belonging to the superfamily of P-type ATPases [10]. In *Arabidopsis*, PM HAs are encoded by 11 *HA* genes, among which *H^+^-ATPase 2* (*AHA2*) plays a key role in rhizosphere acidification through H^+^ extrusion in roots upon iron deficiency [11]. In apple, *MdAHA8* is one of the closest homologs to *Arabidopsis AHA2*, and its transcript level was significantly upregulated in response to iron deficiency; overexpression of *MdAHA8* in apple calli (instead of roots due to the difficulty of apple transformation) promoted H^+^ excretion of the calli upon iron-deficiency treatment [12]. In cucumber, likewise, transcript expression of the *HA* gene *CsHA1* was also induced in roots by iron deficiency [13]. It is worth mentioning that, though the importance of PM HAs in iron uptake has been proposed, their exact biological function in roots has not yet been evidenced by genetic manipulation in crops, except for the model plant *Arabidopsis*.

The transcription level of genes involved in iron uptake was reported to be tightly regulated by numerous basic helix-loop-helix (bHLH) transcription factors (TFs) [1]. In *Arabidopsis*, five bHLH TFs (bHLH 121/34/104/105/115) form heterodimers and positively regulate a core TF ‘FER-LIKE IRON DEFICIENCY-INDUCED TRANSCRIPTION FACTOR (FIT)’-based complex (together with bHLH 38/39/100/101 or POPEYE). Subsequently, the FIT heterodimer activates the expression of hundreds of genes, such as *FRO2* (FERRIC REDUCTION OXIDASE 2, crucial for step 2 of iron uptake in Strategy I plants) and *IRT1* (IRON-REGULATED TRANSPORTER 1, step 3) to enhance iron absorption [14, 15]. Since an excess iron level is toxic to plants, the cytosolic iron concentration must be strictly regulated [16]. Correspondingly, upon excess iron stress, a set of bHLH IVa TFs (bHLH 18/19/20/25) were reported to promote FIT degradation and antagonize the activity of its partners (bHLH 38/39/100/101). Simultaneously, the POPEYE TF acts as a transcription repressor to downregulate the transcription of *FRO2* and *IRT1* genes [17, 18]. Compared with *FRO2* and *IRT1*, the transcriptional regulation of HAs for rhizosphere acidification (step 1) is still elusive. Although a bHLH104 TF was reported to regulate rhizosphere acidification under iron-deficient conditions in *Arabidopsis* [19] and its ortholog could bind the promoter of the *HA8* gene to enhance H^+^ efflux in response to iron deprivation in apple calli [12], direct genetic evidence has not yet been provided to demonstrate the transcriptional regulation of *HA* genes for rhizosphere acidification in roots.

In this study, we used an iron-poor-soil-tolerant citrus species, Zhique (ZQ) [20], and an iron-poor-soil-sensitive citrus species, trifoliate orange (TO), as materials to explore the possible mechanism underlying iron uptake in citrus roots, by comparing the differences in rhizosphere acidification (step 1), iron reduction (step 2) and iron transport (step 3) between the two citrus genotypes. We revealed that an iron deficiency-induced *HA* gene, *HA6*, was involved in rhizosphere acidification and iron uptake. Moreover, the transcription expression of *HA6* was regulated by a transcription factor ,MYB308, which existed exclusively in the tolerant citrus genotype ZQ. This study provides direct genetic evidence to demonstrate the roles of HA in iron uptake and its transcription regulation in citrus, highlighting a potential for genetic manipulation to enhance iron-uptake efficiency in citrus and other crops.

## Results

### H^+^ efflux for rhizosphere acidification is significantly higher in the roots of Zhique than trifoliate orange

**Figure 1:**
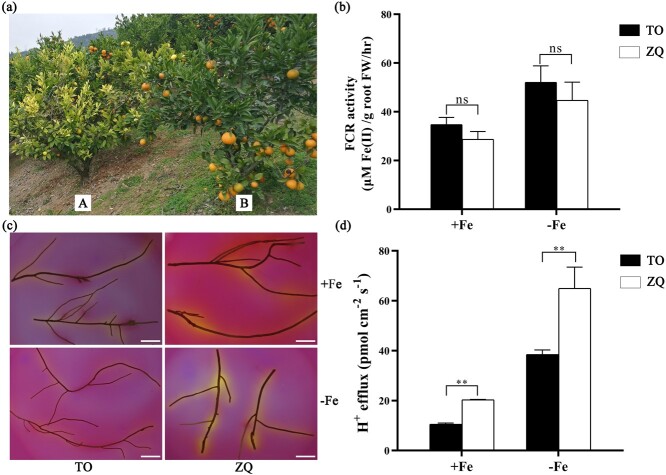
Root acidification and H^+^ efflux of ZQ and TO. **a** ‘Miyagawa Wase’ satsuma mandarin grafted on trifoliate orange (A, TO) rootstock and ‘Zhique’ (B, ZQ) rootstock, respectively. **b** FCR activity in roots of TO and ZQ under +Fe (50 μM Fe) or −Fe (0 μM Fe) conditions. **c** Rhizosphere acidification of TO and ZQ seedlings grown under +Fe or −Fe conditions for 100 days. Acidification is indicated by yellow color around the roots. Scale bar = 2 cm. **d** H^+^ efflux in root hair zone in the root tip of TO and ZQ under +Fe or −Fe treatments. Values are mean ± standard deviation of biological replicates (*n* = 3) ^**^P < .01; ns, not significant (Student’s *t*-test).

Iron absorption of citrus plants belongs to Strategy I (reduction-based strategy), which comprises three steps: (i) rhizosphere acidification; (ii) reduction of Fe^3+^ to Fe^2+^; and (iii) subsequent Fe^2+^ transport into root cells [21]. In calcareous soils, leaves of mandarin grafted on ZQ rootstock were green and the plants generated good fruit yield, while those grafted on TO rootstock showed obvious chlorosis with few fruits (Fig. 1a). Our previous study demonstrated that the chlorosis phenotype was caused by iron deficiency in citrus plants [20, 22]. To explore the differences in iron acquisition between the two citrus rootstock species (ZQ and TO), we first investigated the activity of ferric chelate reductase (FCR), responsible for iron reduction, upon iron-deficiency treatment for 1 week, since iron reduction (step 2) has been reported as the rate-limiting step for iron uptake [23]. The results show that FCR activity is slightly lower in ZQ than TO, though no significant difference was found (Fig. 1b). Correspondingly, the transcription expression of *FRO2* was lower in ZQ than in TO (Supplementary Data Fig. S1a). Similarly, the expression of *IRT1*, a key transporter delivering rhizosphere Fe^2+^ into root cells (step 3), was also lower in ZQ than in TO (Supplementary Data Fig. S1b). Obviously, the results from step 2 or 3 could not explain why ZQ has a much stronger iron uptake than TO. We subsequently investigated the rhizosphere acidification (step 1) capacity of ZQ and TO. We grew plants on iron-sufficient media (pH 6.0) for 7 days, transferred them to iron-deficient media (pH 6.0) for 4 days, and then placed them on agar plates containing the pH indicator bromocresol purple for 1 day. Compared with TO, the roots of ZQ exhibited a visible increase in rhizosphere H^+^, which caused the media around the roots to become yellow, regardless of iron treatment (Fig. 1c). Further quantitative detection using non-invasive micro-test technology revealed that the H^+^ efflux rate in the root hair zone of ZQ was significantly higher than that of TO under both normal and iron-deficiency conditions (Fig. 1d).

### Citrus *HA6*, responsible for H^+^ efflux, showed higher transcription expression in Zhique than trifoliate orange

**Figure 2:**
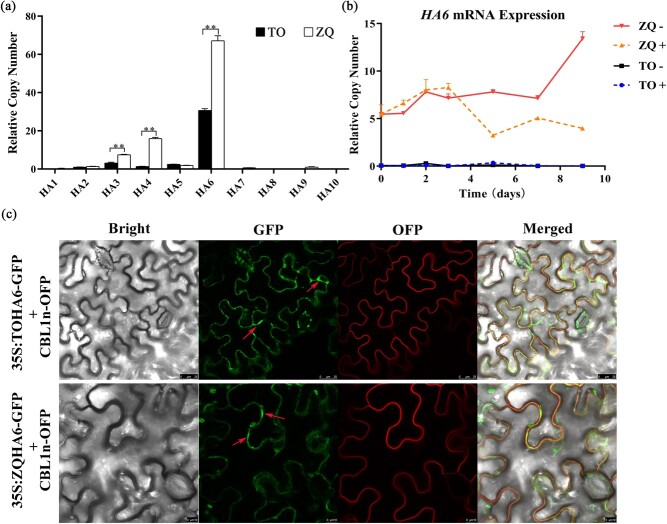
Expression patterns and subcellular localization of *HA6* in TO and ZQ. **a**, **b** Relative abundance of mRNA for 10 *HA* genes (**a**) of the plasma membrane *HA* family and *HA6* gene (**b**) in TO and ZQ under +Fe or −Fe treatment. Values are mean ± standard deviation of biological replicates (*n* = 3). ^**^P < .01 (Student’s *t*-test). **c** Subcellular localization of TOHA6 and ZQHA6 proteins in *N. benthamiana* leaves. CBL1n-OFP was used as a PM marker. Scale bars = 25 or 10 μm.

The PM HAs establish and maintain an H^+^ gradient through pumping H^+^ out of the rhizodermic cells, a process that could be induced by iron deficiency [11]. In citrus, the PM *HA* gene family contains 10 members [24], which are named *HA1* to *HA10* in this study. To investigate which HA members are involved in iron uptake, firstly we determined the steady-state mRNA levels of the 10 *HA* genes in roots under iron-deficiency treatment for 100 days. Notably, *HA6*, *HA4*, and *HA3* showed high expression in ZQ and/or TO, while the other genes (*HA1*, *HA2*, *HA5*, *HA7*, *HA8*, *HA9*, and *HA10*) were almost undetectable under iron deficiency (Fig. 2a). Secondly, a short period (0–60 minutes) of iron-deficiency treatment was then applied to investigate the expression of the three genes (*HA6*, *HA4*, *HA3*). Compared with the control (iron-deficiency treatment for 0 minutes), *HA3* or *HA4* generally showed only 1- to 2-fold expression in both ZQ and TO (Supplementary Data Fig. S2a, b, d, and e), whereas *HA6* showed 2000- to 3000-fold expression in ZQ (Supplementary Data Fig. S2f) but only 0- to 100-fold expression in TO (Supplementary Data Fig. S2c) under iron-deficiency treatment for 15, 30, and 60 minutes. Thirdly, we constructed a phylogenetic tree using the 10 citrus *HA* genes and those from *Arabidopsis*, and found that Cs4g03700.1 (*HA6*) was a putative ortholog that was closest to *AHA2* (Supplementary Data Fig. S3), an *Arabidopsis* gene responsible for rhizosphere acidification under iron deficiency [11]. Together, our results indicated that the highly expressed *HA6* is an ideal candidate that may play a regulatory role in H^+^ efflux in citrus roots under low iron conditions. In line with this idea, the mRNA expression of *HA6* was significantly higher in the roots of ZQ than in TO under iron-deficiency treatment (Fig. 2a and b). These results support our hypothesis that differential expression of *HA6* may cause the difference in rhizosphere acidification between the two citrus rootstock genotypes under iron-starvation conditions.

### Citrus HA6 mainly localizes on the plasma membrane

Citrus HA6 is proposed to localize on the plasma membrane due to its role in secreting intracellular H^+^ into the apoplast to energize nutrient uptake. To confirm the hypothesis, the coding sequence (CDS) of *TOHA6* and *ZQHA6* was respectively cloned from TO and ZQ, green fluorescent protein (GFP) was fused to the C terminus of TOHA6 (TOHA6-GFP) and ZQHA6 (ZQHA6-GFP), and they were co-expressed with the plasma membrane marker protein (CBL1n-OFP) in *Nicotiana benthamiana* leaves. As expected, though there are two amino acid differences between TOHA6 and ZQHA6 proteins, and the green fluorescence signals from both TOHA6-GFP and ZQHA6-GFP were well colocalized with those from CBL1n-OFP (Fig. 2c; Supplementary Data Fig. S4); this means that TOHA6 and ZQHA6 have similar functions. Slight GFP signals were also detected in the cytoplasm (red arrows in Fig. 2c), which might due to the proteins needing to be transported by the cytoplasm secretory pathway to the plasma membrane [25].

### The citrus *HA6* gene could recover the phenotype of the *Arabidopsis aha2* mutant upon iron deficiency

**Figure 3:**
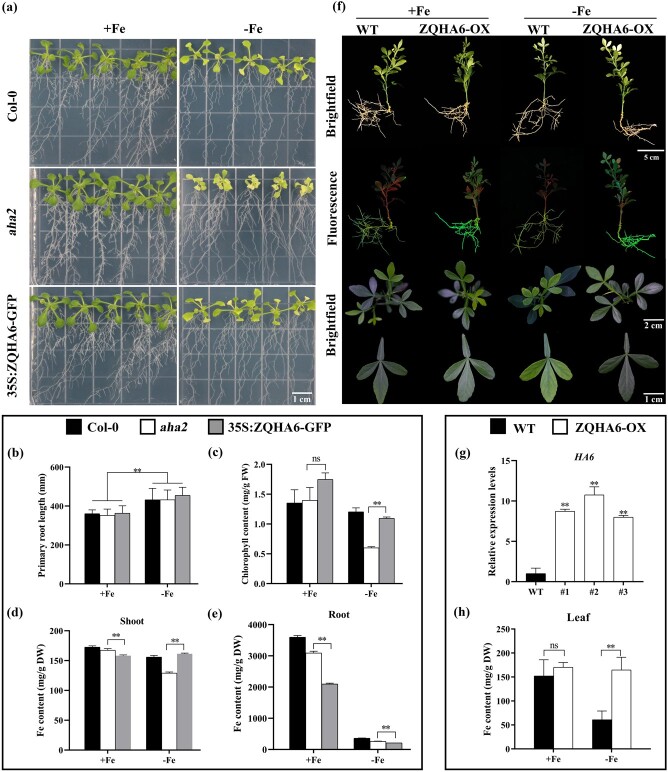
Phenotype of *ZQHA6* transgenic plants under +Fe and −Fe conditions. **a**–**e** Phenotypes (**a**), primary root length (**b**), chlorophyll content (**c**), Fe contents in shoot (**d**) and root (**e**) of *Arabidopsis* WT (Col-0), the *aha2* mutant, and *35S:ZQHA6-GFP* transgenic lines grown on +Fe or −Fe medium for 3 weeks. Scale bar = 1 cm. FW, fresh weight; DW, dry weight. **f** Phenotypes of the TO WT (WT) and transgenic lines *35S:ZQHA6* (ZQHA6-OX) grown on +Fe or −Fe sand media for 28 days. **g** Expression level of the *HA6* gene in WT and ZQHA6-OX. **h** Iron contents of WT and ZQHA6-OX grown on +Fe or −Fe sand media for 28 days. Values are mean ± standard deviation of biological replicates (*n* = 6 in the *Arabidopsis* analysis and *n* = 3 in the citrus analysis). ^**^P < .01; ns, not significant (Student’s *t*-test).

To confirm the biological function of the citrus *HA6* gene in iron uptake, we did a genetic complementary analysis by using an *Arabidopsis aha2* mutant that possesses an obviously lower H^+^ flux under either iron-sufficient (+Fe) or iron-deficient (−Fe) conditions [11]. Here, we cloned the CDS of *HA6* from ZQ, made a *35S:ZQHA6-GFP* construct, stably transformed it in the *aha2* mutant, and obtained a total of 10 transgenic lines with GFP signals in root tips (Supplementary Data Fig. S5). Subsequently, the *Arabidopsis* Col-0 wild-type (WT), *aha2* mutant, and *35S:ZQHA6-GFP* transgenic complementary lines were grown in medium for 3 weeks under +Fe or −Fe conditions (Fig. 3a). The root length of all the plants showed no difference under either +Fe or −Fe conditions (Fig. 3b). Under the +Fe condition, all three genotypes grew well in the medium, with similar chlorophyll contents (Fig. 3a and c). Under the −Fe condition, as expected, the *aha2* mutant showed an obvious chlorosis phenotype with only 0.60 mg/g fresh weight chlorophyll content, whereas the WT and *35S:ZQHA6-GFP* lines displayed normal growth with chlorophyll contents of 1.20 and 1.09 mg/g fresh weight, respectively (Fig. 3a and c). These results indicated that the citrus *HA6* gene could recover the phenotype of the *aha2* mutant under iron-deficiency conditions.

To confirm the phenotype result, we tested iron content in the root and shoot tissues of the three genotypes. Consistent with the phenotype, in the shoots, under the +Fe condition all the plants showed a high iron content, while under the −Fe treatment only *aha2* displayed decreased iron levels as compared with Col-0 and *35S:ZQHA6-GFP* (Fig. 3d). Unexpectedly, in the roots and shoots, the iron content of *35S:ZQHA6-GFP* plants was significantly decreased compared with Col-0 and *aha2* under the +Fe condition (Fig. 3d and e). Likewise, the iron contents showed a significant decrease in transgenic roots compared with Col-0 and *aha2* under −Fe treatment (Fig. 3e). The Mg and Zn contents were largely increased in *35S:ZQHA6-GFP Arabidopsis* roots/shoots under the +Fe condition (Supplementary Data Fig. S6). However, under the −Fe condition, the Mg and Zn contents were slightly increased in shoots and were not changed in roots of *35S:ZQHA6-GFP Arabidopsis* plants (Supplementary Data Fig. S6).

### Overexpression of the *HA6* gene in roots significantly increases the iron content of trifoliate orange

To further demonstrate the role of *HA6* in iron uptake of citrus roots, we made a *35S:ZQHA6* (ZQHA6-OX) construct and stably transformed it into TO, resulting in six transgenic lines with significant GFP signals and increases in *HA6* transcription (Fig. 3f and g). In ZQHA6-OX transgenic plants, the expression of *FRO2* was also upregulated, while the expression of *IRT1* was not affected (Supplementary Data Fig. S7a and b). Subsequently, the WT and ZQHA6-OX transgenic lines were grown in sand media for 28 days under +Fe or −Fe conditions. Under the +Fe condition both the WT and transgenic plants grew well in the medium, and under the −Fe treatment the transgenic plants grew well, whereas the WT showed obvious interveinal chlorosis in young leaves (Fig. 3f). Consistent with the phenotype, in the leaves, under the +Fe condition the WT and ZQHA6-OX transgenic lines showed no difference in iron content, while under the −Fe treatment the ZQHA6-OX transgenic plants displayed increased iron levels as compared with WT plants (Fig. 3h). We also determined the contents of Mg and Zn (both associated with photosynthesis) in the leaves and roots. In leaves, under the +Fe treatment the content of either Mg or Zn in the ZQHA6-OX transgenic lines showed no difference from WT, and under the −Fe treatment a slight increase was only found in the Mg content of the transgenic lines as compared with WT (Supplementary Data Fig. S8a and b). In roots, under the +Fe condition, overexpression of *ZQHA6* resulted in an increase in Zn content and a decrease in Fe content (Supplementary Data Fig. S8c–e), but under the −Fe condition the Fe, Mg, and Zn contents showed no change in ZQHA6-OX as compared with WT (Supplementary Data Fig. S8c–e).

### Transcription of *HA6* is activated by transcription factor MYB308 in Zhique but not in trifoliate orange

**Figure 4:**
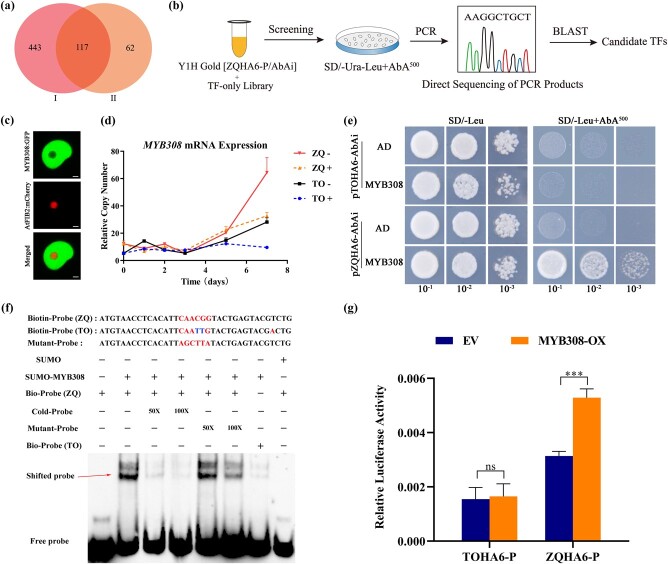
MYB308 directly activates the expression of the *ZQHA6* gene. **a** Venn diagrams of TFs in two databases (I, TFs searched by RNA-seq; II, TFs predicted on the PlantRegMap website). **b** Flowchart for screening TFs that bind to the *ZQHA6* promoter. **c** Subcellular localization of MYB308 protein. MYB308-GFP was co-transformed with the nucleolus marker AtFIB2-mCherry into *N. benthamiana* leaves. Scale bar = 10 μm. **d** Relative abundance of mRNA for the *MYB308* gene in TO and ZQ under +Fe and −Fe treatments. **e** Y1H assay showing that MYB308 bound to the promoter of *ZQHA6* but not *TOHA6*. The assay was performed three times with the same result. **f** MYB308 bound to the motifs of the *ZQHA6* promoter *in vitro*, as indicated by an EMSA method. +, presence −, absence. **g** MYB308 activated the expression of *ZQHA6* but not *TOHA6*. The firefly LUC/*Renilla* LUC ratio represents the relative activity of the *ZQHA6* and *TOHA6* promoters. Values in each column are mean ± standard deviation of biological replicates (*n* = 3 in gene expression analysis and *n* = 6 in LUC analysis). ^***^P < .001; ns, not significant (Student’s *t*-test).

To explore the mechanism underlying the differential expression of *HA6*, we cloned the promoters of *HA6* from ZQ and TO and found there were 48 SNPs (single-nucleotide polymorphisms) and 15 InDels (small insertions and deletions) between the promoter sequences of two citrus genotypes (Supplementary Data Fig. S9), suggesting that promoter variation may be responsible for the differential transcription of *HA6* in citrus roots. As promoters were activated by TFs, we aimed to isolate TFs that might interact with the *cis*-regulatory elements (CREs) of the *HA6* promoter. A total of 561 TFs were found in the transcriptome of ZQ and TO (Supplementary Data Table S3), and bioinformatic analysis predicted that 117 out of the 561 TFs probably bind the promoter of *HA6* gene (Fig. 4a). The CDSs from all the 117 TFs were cloned into a pGADT7 vector and thus we obtained a TF library, and then the library was screened using yeast harboring the *ZQHA6* promoter sequence, leading to a positive colony that contain the CDS of MYB308 (Fig. 4b; see Materials and methods). Since the MYB308 CDS showed the same sequences between ZQ and TO (Supplementary Data Fig. S10), only the MYB308 cloned from ZQ was used for subsequent analysis. As a transcription factor, the MYB308 was confirmed to localize to the nucleus by co-expression with a nucleus marker protein, AtFIB2-mCherry, in tobacco leaves (Fig. 4c). Interestingly, the mRNA expression of MYB308 was significantly higher in the roots of ZQ than in TO under iron-deficiency treatment (Fig. 4d).

A yeast one-hybrid (Y1H) experiment confirmed that the MYB308-GAL4 could bind the promoter of *ZQHA6* in yeast (Fig. 4e), which suggests that the MYB308 binds the *ZQHA6* promoter *in vivo*. However, MYB308-GAL4 could not bind the promoter of *TOHA6* (Fig. 4e). An electrophoretic mobility shift assay (EMSA) was also performed to validate the binding of MYB308 and *HA6* promoter *in vitro*. Since MYB308 is predicted to be a member of the R2R3-MYB family, which could bind the MBS type I sequence [C(A/C/G/T)GTT(A/G)] [26], we first searched the *ZQHA6* promoter using the motif and found a reverse complementary sequence (CAACGG) located in the *ZQHA6* promoter, which is different from the *TOHA6* promoter (see red box in Supplementary Data Fig. S9). An EMSA was thus conducted to validate the binding of MYB308 and a 36-bp biotin-labeled probe containing the (CAACGG) motif. The results showed that the MYB308 could bind to the *ZQHA6* promoter while it lost binding activity when the binding motif was mutated (Fig. 4f). The motif (CAATTG) of the *TOHA6* promoter has two-base variation (the underline indicates the difference) with that of the *ZQHA6* promoter, and the MYB308 has only very weak binding with the *TOHA6* promoter (Fig. 4f). The regulatory role of MYB308 for *HA6* expression was further investigated by the luciferase (LUC) reporter assay. Firefly LUC reporter constructs driven by *ZQHA6* or *TOHA6* promoter were created, and an effector construct was generated using MYB308. Our results showed that co-expression of the effector with the *ZQHA6* reporter activated the LUC >2-fold relative to the control (Fig. 4g), while *TOHA6* reporters showed no change, indicating that MYB308 could function as a transcriptional activator of *ZQHA6* but not *TOHA6* (Fig. 4g).

**Figure 5:**
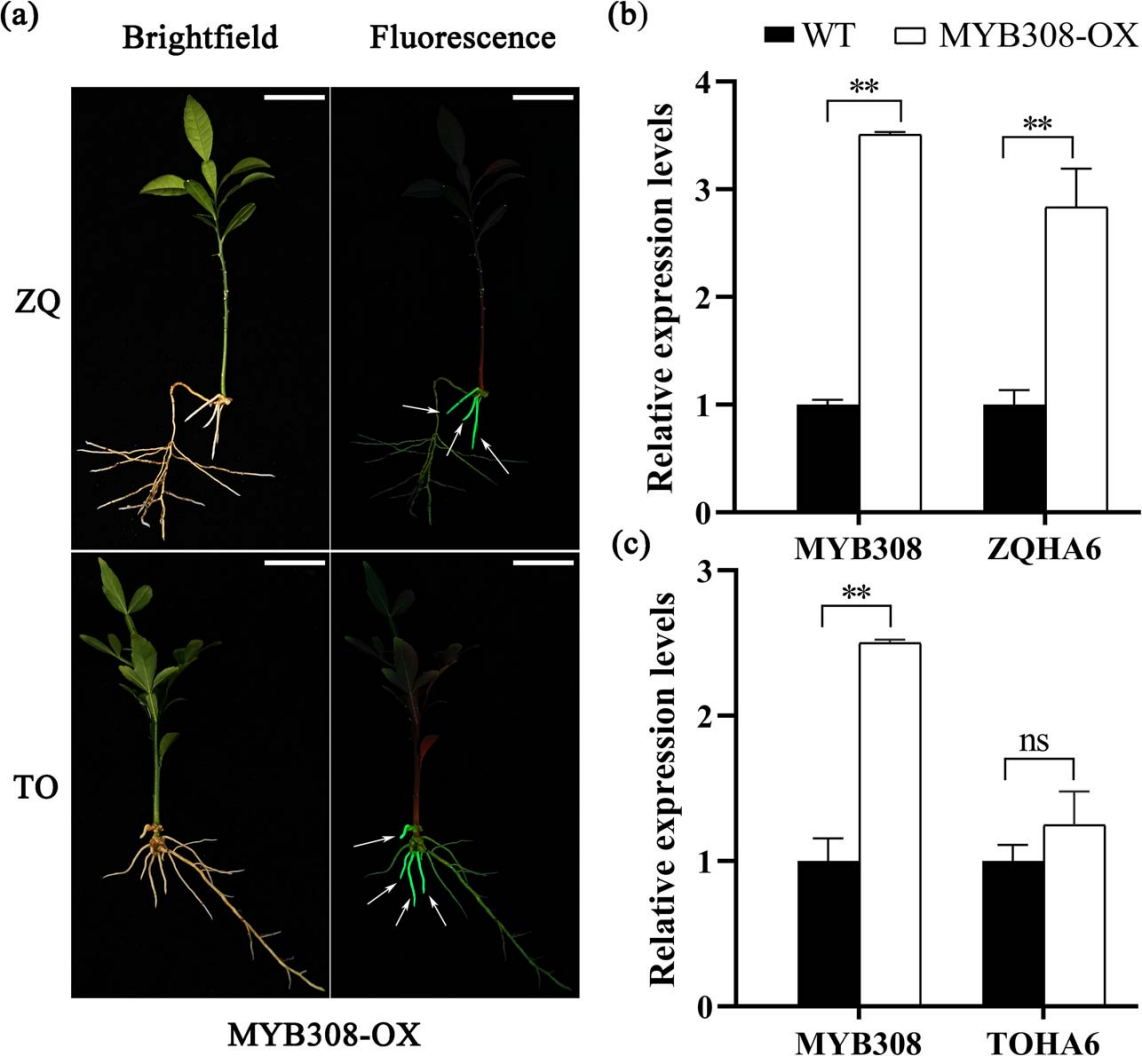
Expression levels of *ZQHA6* and *TOHA6* in MYB308 transgenic lines. **a** Phenotypes of the MYB308-OX transgenic lines of ZQ or TO grown on +Fe (50 μM Fe) sand media for 3 weeks. Arrows indicate MYB308-OX transgenic roots; roots of the same plant without GFP signals were used as control (WT). Scale bar = 2.5 cm. **b**, **c** Relative expression of *MYB308* and *HA6* in roots of ZQ (**b**) and TO (**c**). Values in each column are mean ± standard deviation of biological replicates (*n* = 3). ^**^P < .01; ns, not significant (Student’s t-test).

To further validate the putative function of MYB308 in the transcription of the *HA6* gene in citrus roots, we performed stable overexpression of *MYB308* (MYB308-OX) in hairy roots of both ZQ and TO, and the transgenic-positive roots were selected by observing GFP signals (arrows in Fig. 5a). As expected, under the +Fe condition the transcription of *MYB308* was significantly higher in the transgenic roots of ZQ or TO than in WT (Fig. 5b and c). Correspondingly, the transcription of *HA6* was highly induced in ZQ transgenic lines, but it showed no change in TO transgenic lines (Fig. 5b and c). The results suggest that MYB308 could activate the transcription expression of *HA6* gene in ZQ but not TO.

### Genetic diversity and evolution of *HA6* promoters in different citrus species

**Figure 6:**
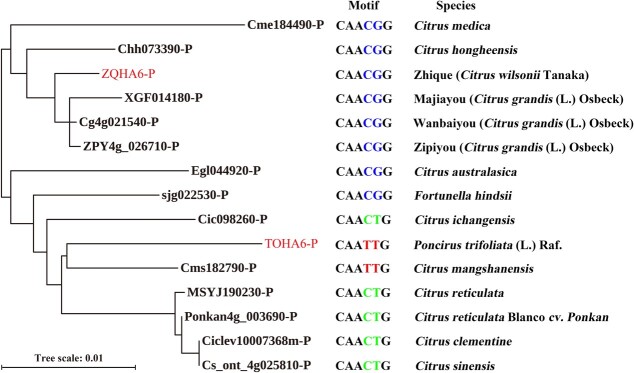
Phylogenetic tree of *HA6* promoters in different citrus species. The MYB308 binding motif (CAACGG) in ZQ and pummelo are marked in blue, and its variants in old citrus species (CAATTG) and mandarin-type citrus (CAACTG) are marked in red and green, respectively.

To investigate the genetic diversity and evolution of the *HA6* promoter sequences, we obtained and analyzed the promoter sequences of *HA6* genes from ZQ, TO, the old citrus species *Citrus mangshanensis*, and 12 citrus cultivars from Citrus Genome Data (http://citrus.hzau.edu.cn/). The MYB308 binding motif (CAACGG) of the *HA6* promoters were found to exist in ZQ and pummelo species, while its variants CAATTG and CAACTG, which cannot bind MYB308, were found to exist in the old citrus species *C. mangshanensis* and TO, and mandarin species, respectively (Fig. 6). Therefore, the sequence motif may experience two mutation events (CAATTG → CAACTG → CAACGG) during citrus evolution and ultimately lead to the MYB308 binding motif CAACGG in ZQ.

## Discussion

### HA6 is an H^+^-ATPase required for rhizosphere acidification and iron uptake

Due to the sedentary lifestyle, plants have evolved mechanisms to acclimate to various environmental stresses, including soil nutrient deficiency [27]. The soil rhizosphere nutrients are absorbed by roots of plants through a set of transporters localized on the plasma membrane of root epidemic cells. Among these, the proton pump HAs are transporters responsible for establishing the electrochemical gradient involved in membrane energization for solute transport, and thus they play important roles in root nutrient uptake and xylem or phloem loading [10].

Iron is a key nutrient for plants due to its roles in many cellular functions, such as photosynthesis and respiration [28]. The acidification of the rhizosphere to solubilize Fe^3+^ complexes from soil was considered to be a prerequisite for iron uptake in Strategy I plants [29]. The H^+^ extrusion mediated by PM HA was found to be a major factor that leads to rhizosphere acidification and increases root capacity to mobilize and acquire iron from soil [30, 31]. Therefore, the activation of PM HA is considered important for iron acquisition in plants.

The present study reports a specific *HA6* gene in citrus that is one of the closest homologs among 10 citrus PM HAs to *AHA2*, a PM *HA* gene member crucial for rhizosphere acidification and iron uptake in *Arabidopsis* (Supplementary Data Fig. S3). Similar to *AHA2*, the transcription of citrus *HA6* was highly induced under iron deprivation and its encoding proteins mainly localized on the PM; moreover, overexpression of *HA6* in an *aha2* mutant background recovered the WT phenotype in *Arabidopsis* (Fig. 3a), suggesting that HA6 is an HA responsible for H^+^ extrusion and rhizosphere acidification in citrus. Under the +Fe condition, the Mg and Zn contents were also increased in *35S:ZQHA6-GFP Arabidopsis* roots/shoots (Supplementary Data Fig. S6), indicating that ZQHA6 possibly could also promote Mg and Zn absorption. In turn, the enrichment of such divalent metal cations might antagonize Fe uptake [32], leading to a decrease in Fe content as compared with WT/*aha2* under sufficient Fe supply (Fig. 3d and e). However, under the −Fe condition, the Mg and Zn contents were only slightly increased in shoots and were not changed in roots of *35S:ZQHA6-GFP Arabidopsis* plants (Supplementary Data Fig. S6). Therefore, no antagonistic effect between the divalent metal cations was observed under the −Fe condition. Additionally, the slight decrease in Fe content in roots of *35S:ZQHA6-GFP Arabidopsis* plants under the −Fe condition was possibly due to the transfer of Fe from roots to shoots to maintain photosynthesis in the plants (Fig. 3d and e).

To identify the biological function of *HA6* in citrus plants, we used hairy root transformation technology to create *ZQHA6* overexpression roots and discovered that *ZQHA6* may play a strong role in the uptake not only of Fe but also of other mineral elements, such as Mg and Zn; however, it could play a preferential role in Fe absorption under Fe-deficiency stress (Fig. 3h, Supplementary Data Fig. S8). Though the function of HAs in rhizosphere acidification and iron uptake has been investigated by phenotyping the *aha2* mutant in *Arabidopsis* [11] and by overexpressing *MdHA8* in apple calli [12], our study provided complementary genetic evidence to demonstrate the role of PM HA, as the homologous expression of *HA6* was performed in citrus roots where the H^+^ extrusion occurs.

### MYB308 is a transcription factor for *HA6* gene expression in citrus

To explore the mechanism underlying the transcription regulation of the *HA6* gene, we used the Y1H method to screen a TF library, and revealed that the MYB308 TF binds to the promoter of *HA6*, and this was followed by further EMSA and LUC validation (Fig. 4e–g). In this study, to obtain a comprehensive TF library, we generated a transcriptome of the citrus roots under field conditions and then selected those 117 TFs predicted to bind the *HA6* promoter for library construction (Figs 1a and 4a and b). Therefore, the library is believed to cover all the possible TFs regulating *HA6* gene expression.

Plant MYB TFs belong to a big gene family implicated in various responses to biotic/abiotic stresses, such as iron nutrient deficiency [26]. Overexpression of an orchid R2R3-MYB gene, *DwMYB2*, in *Arabidopsis* confers iron accumulation in roots by regulating iron uptake-associated genes such as *FRO2* and *IRT1* [33]. In *Arabidopsis*, *MYB10* and *MYB72* act in the iron-deficiency regulatory cascade to drive gene expression of *NAS4* (NICOTIANAMINE SYNTHASE 4) and thus are essential for plant survival under iron deficiency [34]. Here, we demonstrate that iron deficiency induced MYB308 to bind the ZQ*HA6* promoter (Fig. 4e and f) and positively regulated the expression of ZQ*HA6* in both the tobacco system (Fig. 4g) and citrus roots (Fig. 5b). Therefore, we propose that MYB308 could drive the expression of *HA6* to activate H^+^ excretion and rhizosphere acidification for iron absorption.

In citrus, MYB308 was reported to negatively regulate lignification of citrus fruit juice sacs by acting as a transcriptional repressor of a lignin biosynthetic gene, *4CL* (4-coumarate:CoA ligase) [35]. In addition, lignin accumulation in citrus juice sacs was accompanied by a decline in the activity of HA [36]. These results implied that, besides playing a role in iron uptake, MYB308 might also be involved in lignin biosynthesis by regulating HA activity in citrus, though whether there is any crosstalk between iron uptake and lignification is unknown.

### Possible mechanism of the tolerance of iron deficiency in citrus species Zhique

Compared with the citrus species TO, a commonly used rootstock in citrus cultivation, the citrus species ZQ showed strong tolerance of iron deficiency in calcareous soil conditions [20, 22]. Our results showed that under iron deficiency the transcription expression of *HA6* is much higher in ZQ than TO, thus triggering stronger H^+^ extrusion and rhizosphere acidification in ZQ (Figs 1 and 2). However, the expression of *FRO2* and *IRT1*, which are respectively responsible for iron reduction of Fe^3+^ to Fe^2+^ and Fe^2+^ import, was lower in ZQ than TO (Supplementary Data Fig. S1). This result suggested that, as rhizosphere acidification is strong enough in ZQ, the plants may slow down iron reduction and import to avoid over-absorbing Fe^2+^ in ZQ. Moreover, the TF MYB308 could bind the promoter of *ZQHA6* but not *TOHA6* (Fig. 4), and correspondingly overexpression of MYB308 in ZQ increased the transcript level of *ZQHA6*, whereas in TO *TOHA6* showed no change (Fig. 5b and c). Additionally, under iron deficiency MYB308 expression is high in ZQ but relatively low in TO based on transcription analysis (Fig. 4d). Taken together, the results indicated that the MYB308-mediated high expression of *ZQHA6* might be, at least in part, responsible for the tolerance of iron deficiency in the ZQ species.

The different binding of TFs with promoters is considered to be determined by the nucleotide variation of the promoter sequences [37, 38]. Our results showed that, though the nucleotide similarity of *ZQHA6* and *TOHA6* promoters reaches 92.4%, there are still 48 SNPs and 15 InDels between the two promoters (Supplementary Data Fig. S9). Further sequence and evolution analysis using different citrus species (Fig. 6) suggested that the MYB308 binding motif (CAACGG) in ZQ might experience two mutation events (CAATTG → CAACTG → CAACGG) during acclimation to environment stresses such as alkali-saline stress. Given that MYB308 cannot bind the promoter of *TOHA6* to drive the expression of *TOHA6*, the transcription of *TOHA6* is probably regulated by some other, unknown TFs. Recently a MdbHLH104 transcription factor was reported to regulate an *MdHA8* gene (a homolog of *HA6* here) in apple [12]. It will be interesting to test whether bHLH104 could also regulate the *HA6* gene in citrus and how bHLH104 and MYB308 might differentially or cooperatively regulate the transcription expression of *HA* genes during the iron absorption process in plants.

## Materials and methods

### Plant materials and treatments

Seedlings of Zhique (ZQ, *Citrus wilsonii* Tanaka) and trifoliate orange [TO, *Poncirus trifoliata* L. Raf.] were cultured as described in our previous work [22]. Uniform size seedlings were selected and grown for 30 days in modified Hoagland’s nutrient solution containing either 50 μM Fe(III)-EDTA or 0 μM Fe(III)-EDTA. After 0, 15, 30, and 60 minutes, and 1, 2, 3, 5, 7, and 9 days of iron-deficiency treatments, 2-cm-length root tips from at least three plants were collected and used. ZQ and TO seedlings grown in substrate for 1 year were used for stable hairy root transformation (see section Vector construction and plant transformation). Subsequently, the transgenic plants were transplanted to sand media with three plants in one pot. In the first 2 weeks, all pots were supplied daily with 500 ml of a complete Hoagland’s nutrient solution. The iron-deficiency treatment was conducted as above.

Seeds of *Arabidopsis* T-DNA insertion line *aha2* (SALK_062371.54.50.x) were purchased from the Arabidopsis Biological Resource Center (ABRC). The *Arabidopsis* ecotype Columbia-0 (Col-0, WT) and the *aha2* mutant line (Col-0 background) were used as materials for gene function complementary analyses. The *Arabidopsis* seedlings were cultured as described by Gao *et al*. [39], and then transplanted to Fe-sufficient medium [50 μM Fe(III)-EDTA] or Fe-deficient medium [0 μM Fe(III)-EDTA].

### Visualization of rhizosphere pH and measurement of H^+^ efflux

Citrus rhizosphere acidification was analyzed as described previously [40]. The scanning ion-selective electrode technique was used for measuring H^+^ efflux fluxes [41]. The activity of FCR was measured in whole roots by spectrophotometric measurement of the purple-colored Fe(II)-ferrozine complex [42].

### Multiple sequence alignment and phylogenetic analysis

Citrus and *Arabidopsis HA* gene sequences were downloaded from the sweet orange genome database (http://citrus.hzau.edu.cn/) and Phytozome (https://phytozome-next.jgi.doe.gov/), respectively. Multiple sequence alignments of nucleotide sequences were carried out using MAFFT [43]. The phylogenetic tree was built using the maximum likelihood method available in IQ-TREE with 1000 bootstrap replicates [44]. The Interactive Tree of Life (iTOL; https://itol.embl.de/) was used for visualization of phylogenetic trees.

### Vector construction and plant transformation

Unless indicated, all the constructs were made using the Golden Gate cloning system [45]. All primers and vectors used are listed in Supplementary Data Tables S1 and S2, respectively. The CDSs of HA6 and MYB308 were obtained for ZQ or TO, and then cloned to expression vectors (BF-SXGA or H2gE-35SA). For gene function complementary analysis, the expression vector *35S:ZQHA6-GFP* was carried in *Agrobacterium tumefaciens* strain GV3101 and transformed to the *Arabidopsis aha2* mutant by the floral dip method [46], then the positive lines were selected using the pFAST-R selection cassette [47]. Phenotype analysis was performed using homozygous T2 plants. Citrus stable hairy root transformation was conducted according to the protocol described previously [48]. The transgenic hairy roots were formed ~4 weeks after transformation, and the positive roots were selected by observing GFP.

### Subcellular localization

The *HA6* and *MYB308* CDSs without stop codon were amplified and cloned in SXGA vector. Plasmids were transformed into *A. tumefaciens* GV3101 and transfected to *N. benthamiana* leaves, together with plasma membrane marker CBL1n-OFP or nucleus marker AtFIB2-mCherry [49, 50]. The subcellular localization of HA6 and MYB308 was observed under a confocal laser scanning microscope (TCS SP8; Leica Wetzlar, Germany).

### Determination of iron and chlorophyll content

All citrus and *Arabidopsis* samples were digested according to a previous method [51]. The iron concentrations were determined using an Agilent 5100 SVDV ICP-OES (Agilent Technologies). The chlorophyll content of plant leaves was measured by a spectrophotometric method [52].

### RNA-seq, transcription factor library construction, and yeast one-hybrid assay

Total RNA was extracted from lateral roots of ZQ and TO (three adult trees per genotype in field condition; Fig. 1a), and a total of six RNA-seq libraries were constructed by Novogene (Beijing, China). Gene expression levels were calculated by the method of fragments per kilobase of transcript per million mapped reads (FPKM) using HTSeq [53]. According to the TF annotation in the sweet orange genome database, a total of 561 TFs were identified in the transcriptome of ZQ or TO (FPKM >1; Supplementary Data Table S3). In parallel, a total of 179 TFs were predicted to bind the promoters of *ZQHA6*, *ZQFRO2*, and *ZQIRT1* by using the PlantRegMap website (http://plantregmap.gao-lab.org/binding_site_prediction.php). A total of 117 TFs were found in both TF datasets (561 and 179 TFs), and the 117 TFs were then cloned into pGADT7 to generate a prey with a Smart Assembly Cloning Kit (Smart-Lifesciences, Changzhou, China). Equal amounts of each TF-pGADT7 were pooled and used as the TF library. Promoter fragments of *ZQHA6* or *TOHA6* were incorporated into the pAbAi vector to construct a bait. The Y1H assay was performed as described in the instruction manual (Clontech). The list of TFs is provided in Supplementary Data Table S3.

### Electrophoretic mobility shift assay

The CDS of MYB308 was inserted into a pCold-SUMO plasmid (pCold-MYB308), and transformed into the *Escherichia coli* strain Rosetta (DE3). The MYB308 proteins with SUMO-His tag were purified from the cell extract through an affinity chromatography column filled with resin (Ni NTA Bead 6FF; Smart-Lifesciences, Changzhou, China). The EMSA was carried out with a Light Shift Chemiluminescent EMSA Kit (GS009, Beyotime Biotechnology, China).

### Dual luciferase assay

In order to clarify the transcriptional regulation by MYB308 of the *HA6* gene, the promoter of *HA6* from TO or ZQ was inserted into pGreenII0800-LUC to form the reporter construct *35S:REN*-*pHA6:LUC*. The CDS of *MYB308* was cloned into BE-SXP vector to generate an effector, *35S:MYB308*. Plasmids of *35S:REN*-*pHA6:LUC* and *35S:MYB308* were transformed into *A. tumefaciens* GV3101(pSoup-p19), which was then co-infiltrated into 4-week-old *N. benthamiana* leaves. The relative LUC was assayed using the Dual-Luciferase^®^ Reporter Assay System (Promega, Madison, WI, USA), according to the manufacturer’s instructions. Six *N. benthamiana* plants (six biological replicates) were used here.

### RNA extraction and quantitative real-time PCR

Total RNA extraction and quantitative real-time PCR were performed as we described previously [20]. Data on gene amplification were normalized to the housekeeping gene *Cs1g05000.1* (ΔCt). Relative copy number (RCN) was calculated as follows: RCN = 2^-ΔCt^ × 100, where ΔCt = Ct(target) − Ct(reference). Fold change was quantified with the 2^-ΔΔCt^ method [54].

## Supplementary Material

Web_Material_uhac088

## Data Availability

The RNA-seq datasets generated in this study have been deposited in the Sequence Read Archive (SRA) under the accession number PRJNA791552. Other data supporting our findings are available in the paper and its supplementary information files.
