# Supplementary material for: MYB308-mediated transcriptional activation of plasma membrane H+-ATPase 6 promotes iron uptake in citrus
Source: Hortic Res. 2022 Apr 11;9:uhac088. doi: 10.1093/hr/uhac088 (PMC9171118; doi:10.1093/hr/uhac088)
Supplement: Web_Material_uhac088 [file web_material_uhac088.zip › Supplementary figure.doc]

**Supplementary Fig. S1** Transcription expression levels of *FRO2* and *IRT1* genes in the roots of TO and ZQ seedlings under +Fe or -Fe treatments.

**Supplementary Fig. S2** Transcription expression levels of *HA3*, *HA4*, and *HA6* genes in the roots of TO and ZQ seedlings grown on +Fe or -Fe media for 60 minutes. (n.d. = not detected)

**Supplementary Fig. S3** Phylogenetic tree of plasma membrane HA proteins from citrus and Arabidopsis. (AHA1: AT2G18960; AHA2: AT4G30190; AHA3: AT5G57350; AHA4: AT3G47950; AHA5: AT2G24520; AHA6: AT2G07560; AHA7: AT3G60330; AHA8: AT3G42640; AHA9: AT1G80660; AHA10: AT1G17260; AHA11: AT5G62670)

**Supplementary Fig. S4** Comparison of the amino acid sequences between TOHA6 and ZQHA6 proteins. Different amino acids are labeled with red arrow.

**Supplementary Fig. S5** Confocal microscope image of Arabidopsis roots of T2 plants showing GFP fluorescence as a result of ZQHA6-GFP expression. Bar = 50 μm

**Supplementary Fig. S6** The Mg and Zn contents in shoot or root of the Arabidopsis wild type (Col-0), *aha2* mutant and *35S:ZQHA6-GFP* transgenic lines grown on +Fe or -Fe medium for 3 weeks. DW: dry weight. Values were means ± SD of biological replicates (n = 6 in Arabidopsis analysis) (*P < 0.05, **P < 0.01 with Student's t-test).

**Supplementary Fig. S7** Transcriptional expression levels of the *FRO2* or *IRT1* genes in citrus WT and ZQHA6-OX transgenic plants grown on +Fe sand-media for 14 days. Values were means ± SD of biological replicates (n = 3) (**P < 0.01 with Student's t-test, ns = not significant).

**Supplementary Fig. S8** The Fe, Mg, and Zn contents in shoots or roots of the citrus WT and ZQHA6-OX transgenic plants grown on +Fe or -Fe sand-media for 28 days. Values were means ± SD of biological replicates (n = 3) (*P < 0.05 with Student's t-test, ns = not significant).

**Supplementary Fig.** **S9** Comparison of the promoter sequences between *TOHA6* and *ZQHA6*. Identical nucleotides are indicated in blue background, different nucleotides are labeled in white background. Gaps in the sequences to allow optimal aligning are represented by dots. The binding motif of MYB308 is labeled with red box.

**Supplementary Fig. S10** Comparison of the coding sequences between *TOMYB308* and *ZQMYB308* genes.
